# Impact of Collagen Crosslinking on Dislocated Human Shoulder Capsules—Effect on Structural and Mechanical Properties

**DOI:** 10.3390/ijms23042297

**Published:** 2022-02-18

**Authors:** Pauline Cornette, Ilhem Lilia Jaabar, Vincent Dupres, Jean-David Werthel, Francis Berenbaum, Xavier Houard, Jessem Landoulsi, Geoffroy Nourissat

**Affiliations:** 1 Centre de Recherche Saint-Antoine (CRSA), INSERM, Sorbonne Université, F-75012 Paris, France; pauline.cornette@gmail.com (P.C.); ilhem.jaabar@sorbonne-universite.fr (I.L.J.); francis.berenbaum@aphp.fr (F.B.); xavier.houard@sorbonne-universite.fr (X.H.); 2 Laboratoire de Réactivité de Surface, CNRS, Sorbonne Université, F-75005 Paris, France; jessem.landoulsi@sorbonne-universite.fr; 3 U1019-UMR 9017-CIIL-Center for Infection and Immunity of Lille, Institut Pasteur de Lille, CHU Lille, Inserm, CNRS, Université Lille, F-59000 Lille, France; vincent.dupres@ibl.cnrs.fr; 4 Department of Orthopedy and Traumatology, AP-HP Ambroise Paré Hospital, F-92100 Boulogne-Billancourt, France; jdwerthel@gmail.com; 5 Department of Rheumatology, AP-HP Saint-Antoine Hospital, DMU 3iD, F-75012 Paris, France; 6 Clinique des Maussins, 67 Rue de Romainville, F-75019 Paris, France

**Keywords:** shoulder instability, atomic force microscopy, type I collagen fibrils

## Abstract

Classical treatments of shoulder instability are associated with recurrence. To determine whether the modification of the capsule properties may be an alternative procedure, the effect of crosslinking treatment on the structure and mechanical properties of diseased human shoulder capsules was investigated. Joint capsules harvested from patients during shoulder surgery (n = 5) were treated or not with UV and/or riboflavin (0.1%, 1.0% and 2.5%). The structure and the mechanical properties of the capsules were determined by atomic force microscopy. The effect of treatments on cell death was investigated. Collagen fibrils were well-aligned and adjacent to each other with a D-periodicity of 66.9 ± 3.2 nm and a diameter of 71.8 ± 15.4 nm in control untreated capsules. No effect of treatments was observed on the organization of the collagen fibrils nor on their intrinsic characteristics, including D-periodicity or their mean diameter. The treatments also did not induce cell death. In contrast, UV + 2.5% riboflavin induced capsule stiffness, as revealed by the increased Young’s modulus values (*p* < 0.0001 for each patient). Our results showed that the crosslinking procedure changed the biomechanics of diseased capsules, while keeping their structural organisation unchanged at the single fibril level. The UV/riboflavin crosslinking procedure may be a promising way to preserve the functions of collagen-based tissues and tune their elasticity for clinically relevant treatments.

## 1. Introduction

Instabilities of the glenohumeral joint are the frequent disorders of young sportive patients. Dislocations of the shoulder joint are almost systematically associated with bony and capsulolabral disorders. Anatomic lesions like Bankart, HAGL (humeral avulsion glenohumeral ligament) or mid substance tears of the capsule and labrum are well known and are routinely treated by surgical procedures. Even after having fixed those macroscopic tears, recurrence is frequent [1]. A simple plication or direct repair could not treat the instability efficiently because surgery is acting mostly on the soft tissues involved in the procedure [1]. The modification of the entire capsule then appears as an alternative procedure. Hippocrates first described burns of all the anterior aspect of the shoulder in order to perform a scar tissue to limit anterior translation [2]. Thermal capsulorrhaphy has been developed and used to treat many different types of shoulder instability, but the efficacy of the thermal treatment cannot be confirmed [3]. This thermal shrinkage can also be carried out using radiofrequency or laser devices [4]. Rare but detrimental complications and few relevant clinical studies made this technique less popular [4]. Therefore, there is a real unmet need to efficiently treat shoulder instability.

The frequent recurrence of instability is mainly associated with the modification of the capsulolabral tissue structure caused by repetitive trauma. Rodeo et al. [5]. reported that dislocation modifies the structure of the capsule at the (supra)molecular level, showing significant differences in the characteristics of collagen fibrils (i.e., collagen crosslinks, collagen fibril diameter and density and amino acid composition) between healthy and dislocated shoulder capsules. The joint capsule is indeed mainly composed of fibrils of self-assembled type I collagen with a presumable multiaxial orientation [6]. The mechanical properties of the capsule, particularly the tensile strength, depend on the mean diameter of collagen fibrils and the intermolecular crosslinks between collagen molecules within the fibrils, which prevent the slippage under load. The mechanical behaviour is further tuned by the alignment of these fibrils in the tissue [5]. The anterior dislocation primarily affects the tensile behaviour of the glenohumeral capsule rather than the shear behaviour, and this phenomenon could be caused by failure or rotation of the collagen fibrils and/or the plastic deformation of the matrix [7].

In vivo, type I collagen fibrils form by the self-assembly of collagen triple-helix (with molecular dimensions of ∼300 nm in length and ∼1.5 nm in diameter) [8,9] through a cell-mediated regulation process involving two main processes [10]: (i) intermolecular interactions (hydrophobic interactions, hydrogen bonding, electrostatic interaction) determined by the intrinsic properties of collagen molecules [8,9]; and (ii) covalent bonds, which may be of a chemical nature or mediated by enzymes [10]. The controlled enzyme-assisted crosslinking is achieved by the oxidative deamination of specific lysine residues via lysyl oxidases, yielding lysine-aldehydes. The latter react, then, with an opposing hydroxylysine to form a Schiff base. By contrast, the non-enzymatic crosslinking results in an adventitious reaction between collagen and glucose. These crosslinking processes are tissue specific and provide the strength to collagen fibrils, depending on the mechanical requirements of the tissue (elasticity, stiffness, energy storing, etc.). Improving the mechanical properties of tissues by crosslinking has been used in clinical practice in ophthalmology for the treatment of cornea and sclera disorders for a couple of decades [11]. The principle is to change the degree of crosslinking within the collagen fibrils to modify the mechanical properties of the tissue. This interestingly therapeutic approach may be an alternative way to treat shoulder instability.

In the present study, we investigate the combined effect of UV irradiation and riboflavin, a procedure adapted from crosslinking treatments performed on cornea and sclera disorders [11], on the structure and mechanical properties of diseased shoulder capsules harvested from patients during surgery. Owing to the dimensions of the collagen fibrils and their organization in the tissue, it is crucial to probe the effect of crosslinking at the nanoscale. Capsules were, thus, characterized by atomic force microscopy (AFM), one of the main techniques used to probe the organization of collagen in tissues at the nanoscale [12,13,14,15,16,17,18,19,20]. More precisely, the present investigations aim to (i) better define the structure of the collagen in the capsule, (ii) determine the impact of the crosslinking treatment on collagen fibrils structures and mechanical properties, and (iii) define optimal conditions for clinically relevant experimentation of this crosslinking procedure.

## 2. Results

### 2.1. Increased Capsule Stiffness after Crosslinking Procedure

**Figure 1:**
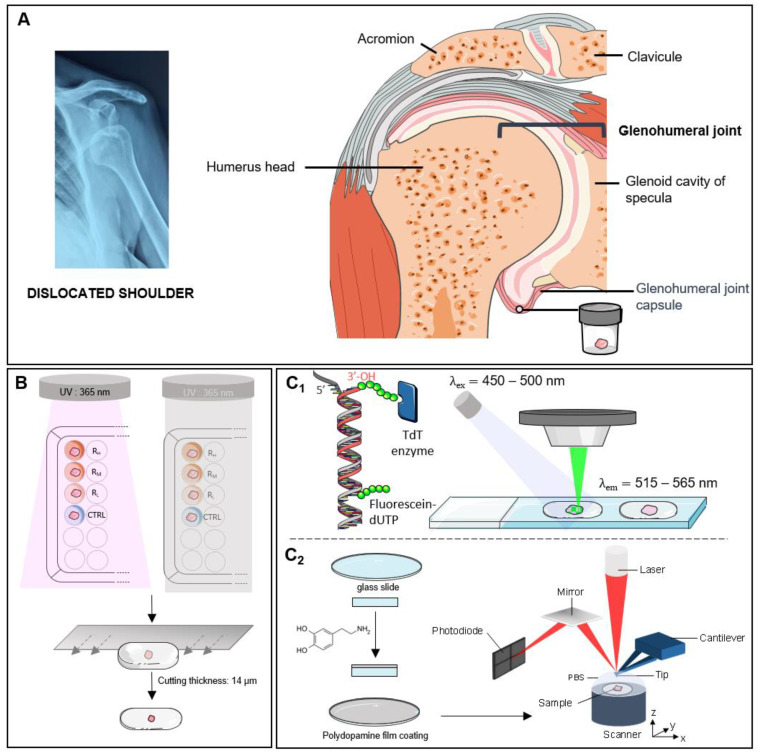
Experimental design. (**A**) Tissue sampling. (**B**) Tissue treatment: Riboflavin treatment (R_H_: 2.5%, R_M_: 1.0%, R_L_: 0.1%) with and without UV radiation, O.C.T. inclusion and subsequent cryosectioning. (**C**) Tissue characterizations: Fluorescent (**C_1_**) for the detection of cell apoptosis and atomic force microscopy (**C_2_**) for probing structural and mechanical properties.

**Figure 2:**
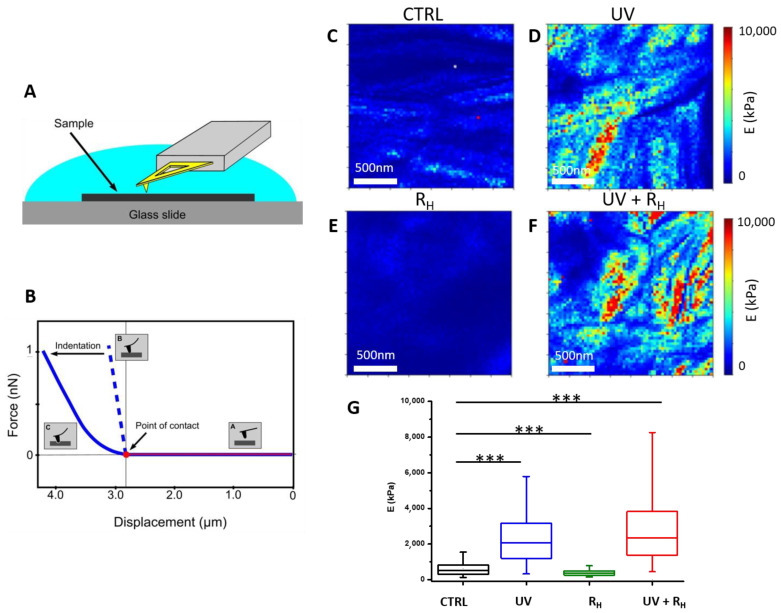
Atomic force microscopy analysis of human capsule elasticity. (**A**) A schematic representation of the experimental set-up. (**B**) Representative force versus displacement curve (the approach part) showing two different behaviours: the probe pushing on a hard surface (dashed line, label B) or indenting in a soft surface (continuous line, label C) (**C**–**F**) Elastic modulus maps (2 µm × 2 µm) obtained for the joint capsule in the different conditions: control (**C**), UV treatment (**D**), 2.5% riboflavin treatment (R_H_) (**E**) and 2.5% riboflavin + UV treatment (UV + R_H_) (**F**). The different colours are representative of lower (blue/green) and higher (red/orange) values of the Young’s modulus. (**G**) Quantification of Young’s modulus obtained by AFM for the joint capsules in the four different conditions for one representative patient (box plots generated by pooling all of the data collected for at least three different elasticity maps per condition and for different regions of the tissue, *** *p*-value < 0.0001).

**Figure 3:**
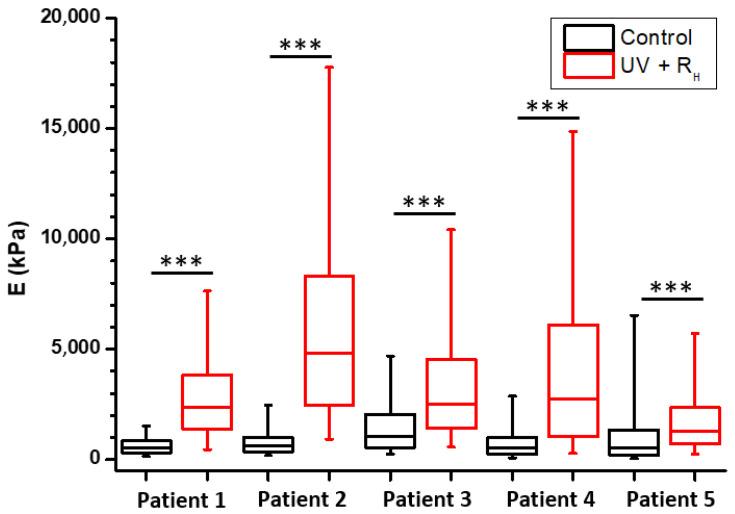
Increase tissue stiffness of human capsules by crosslinking treatment. Comparison of the quantification of Young’s modulus obtained by AFM for the joint capsules in the two different conditions (Control in black and 2.5% riboflavin + UV in red) and for five different patients (box plots generated by pooling all of the data collected for at least three different elasticity maps per condition and for different regions of the tissue, *** *p*-value < 0.0001).

The procedures used for tissue sampling, crosslinking and sample preparation are summarized in Figure 1. AFM was used to compare the mechanical properties of the joint capsules with and without treatment (Figure 2 and Figure 3). To ensure that observed differences between treatment may not be due to an experimental artefact, all experiments for the same patient were always performed using the same AFM tip and by strictly maintaining the same experimental parameters. Figure 2 presents the elasticity maps obtained for one patient in the different experimental conditions, where Young’s modulus is displayed as coloured pixels that reflect the tissue stiffness magnitude. In the control non-treated sample (Figure 2C), the elasticity of the tissue is relatively low compared to UV and UV + 2.5% Riboflavin (UV + R_H_) treatments (Figure 2D,F); the latter treatments induced a strong increase in tissue stiffness (*p* < 0.0001 for both UV and UV + R_H_ treatment) (Figure 2G). By contrast, the riboflavin (R_H_) treatment led to a decrease of the Young’s modulus (*p* < 0.0001) (Figure 2E,G) that may be explained by an effect of riboflavin on the native crosslinking of collagen fibrils. The effects of the treatments on the mechanical properties of the joint capsules observed on this patient reflect the trends obtained for the entire patient panel. Since the most noticeable effect on mechanical properties seems to be related to the UV + R_H_ treatment, and that this treatment is the one expected to induce collagen crosslinking, Figure 3 presents individual comparisons obtained for the five studied patients with the UV + R_H_ treatment. For each patient, an increase in the Young’s modulus after UV + R_H_ treatment was clearly observed. The mean increase in the Young’s modulus values ranged between 1.3 and 7.1-fold (*p* < 0.0001 for each patient) (Figure 3). These results suggest that the crosslinking procedure had a major effect on the elasticity of collagen fibrils within the diseased capsules. Thus, the crosslinking procedure increased the capsules’ tissue stiffness. It is worth noting that box plots also showed high variability on the Young’s modulus values obtained with UV and UV + R_H_ (Figure 2G). This suggests that the treatment induces heterogeneity on the tissue elasticity, which seems to be completely random and did not reveal any anisotropic feature.

### 2.2. Preserved Morphological Characteristics of Capsules and Cell Viability after Crosslinking Procedure

**Figure 4:**
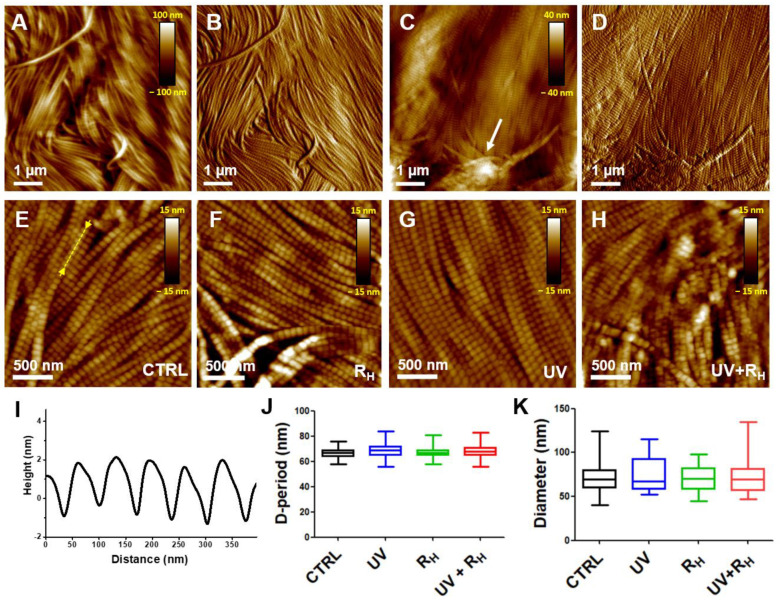
Atomic force microscopy analysis of human capsule structure. Representative AFM (**A**,**C**) height and (**B**,**D**) deflection images (5 µm × 5 µm, contact mode) recorded on untreated joint capsule in (**A**,**B**) PBS buffer or in (**C**,**D**) air. (**E**–**H**) High-resolution images recorded in air of the joint capsule (**E**) prior to and after (**F**) 2.5% riboflavin (R_H_), (**G**) UV or (**H**) 2.5% riboflavin + UV treatments (UV + R_H_). (**I**) Scan line taken at the location indicated by the arrows and dashed line in the image (**E**). (**J**,**K**) Box plots generated by pooling all of the data as a function of the treatment showing the evolution of (**J**) D-period and (**K**) mean diameter of individual fibrils.

Figure 4A,B present AFM images recorded in the hydrated phases (close to physiological conditions) showing the typical morphology of native damaged capsules, i.e., prior to crosslinking treatments. The results showed an alignment of the collagen fibrils. The collagen fibrils are adjacent to each other and exhibit a similar mean diameter. However, the recorded images did not allow the structure of individual fibrils, particularly their D-periodicity, to be sufficiently resolved. This may be due to their mobility during imaging, resulting on their interaction with the AFM tip, as commonly observed on adsorbed collagen layers [21] and collagen-based tissues [22]. In the dried phase (Figure 4C,D), the organization of these fibrils showed an alignment similar to that observed in the hydrated state, even if some disrupted fibrils could be observed (see arrow in Figure 4C). By contrast, the structure of individual collagen fibrils was quite visible, which allowed statistical analyses of their characteristics to be performed in a reliable way.

AFM images were also recorded on capsules treated with UV, R_H_ or with UV + R_H_ (Figure 4F–H). Only the results for high concentration (2.5%) of riboflavin are presented, since similar results were obtained, whatever the concentration of riboflavin used. Results did not show noticeable effects on the whole morphology of the collagen fibrils, i.e., their adjacent and aligned features. Moreover, D-periods remained clearly visible on individual fibrils, regardless of the treatment used.

These qualitative observations suggest that individual collagen fibrils and their organization within the tissue were not greatly affected by the treatments. To obtain quantitative information, the characteristics of collagen fibrils, namely, their mean diameter and D-period, were measured from high-resolution AFM images (Figure 4E–H). For this purpose, at least 100 lines scans were taken from different AFM images, for each treatment, and used to compute D-periodicity. Line scans were taken on individual collagen fibrils along its longitudinal axis. Control non-treated capsules showed well-defined D-periodicity measured at 66.9 ± 3.2 nm (Figure 4E,J), as broadly observed in type I collagen-based tissues [17,18,23]. This is clearly shown through the line scans taken along the fibril longitudinal axis (Figure 4I). Results show that the obtained mean values of the D-period were not affected by R_H_ (67.1 ± 3.1 nm, *p* = 0.0517) and UV + R_H_ (68.2 ± 5.0 nm, *p* = 0.2668) treatment and slightly affected by the UV (68.7 ± 5.3 nm, *p* ≤ 0.001) treatment (Figure 4J). Similarly, the mean values of the fibrils’ diameter were similar in control (71.8 ± 15.4 nm), R_H_ (69.6 ± 14.1 nm, *p* = 0.7090), UV (74.1 ± 18.4 nm, *p* = 0.5940) and UV + R_H_ (74.1 ± 20.7 nm, *p* = 0.9937) treated capsules, confirming the narrow size distribution of fibrils’ diameter in the capsule (Figure 4K).

**Figure 5:**
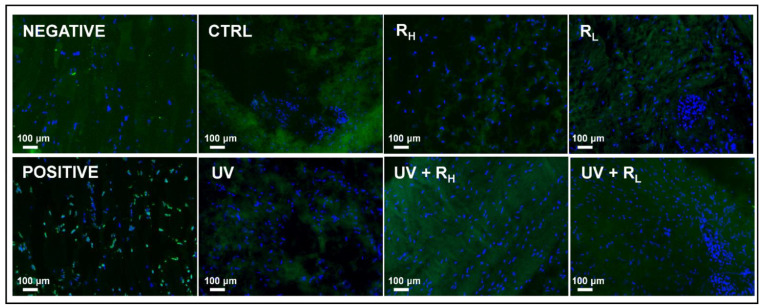
Absence of cell death induction by crosslinking treatment. Human articular capsules were treated or not (CTRL) by UV, 0.1% riboflavin (R_L_), 2.5% riboflavin (R_H_), UV + 0.1% riboflavin (UV + R_L_) or UV + 2.5% riboflavin (UV + R_H_) before TUNEL assay. No cell death was observed in the negative control, whereas many apoptotic cells (green) were present in the positive control. UV and riboflavin alone or in combination did not induce cell death. DAPI staining was used to check the location of the cell nuclei (blue).

Because UV and riboflavin treatments could induce cell death, the presence of apoptotic cells was investigated in treated and non-treated capsules by TUNEL assay (Figure 5). Dead cells appeared as bright green spots as shown in the positive control, whereas blue-fluorescent DAPI is used as nuclear staining. No increase in cell death was observed after treatment by UV, riboflavin, or UV and riboflavin, whatever the concentration of riboflavin used.

These findings clearly indicate that the different treatments, particularly UV + R_H_, did not alter the organization of collagen fibrils within the capsule, i.e., their adjacent and alignment features, and the characteristics of individual collagen fibrils, namely their D-period and mean diameter, nor did they induce apoptosis.

## 3. Discussion

The treatment of shoulder instability by the classical surgery procedure is associated with a high risk of recurrence [1]. In patients with recurrent shoulder instability, the capsulolabral tissue is damaged by repetitive trauma [5]. In the present study, we show that a crosslinking procedure, using riboflavin and UV irradiation, had a major effect on the elasticity of collagen fibrils of human diseased shoulder capsules. The crosslinking procedure reinforced tissue stiffness without altering the characteristics of individual collagen fibrils (namely their D-period and mean diameter) and the organization of these fibrils, without increasing cell death.

Riboflavin was used here as a photosensitizer, which produces radicals within the tissue when exposed to UV irradiation, thus promoting collagen crosslinking. The mechanism by which riboflavin can induce the crosslinking of collagen molecules is described in the work of McCall et al., suggesting the existence of several pathways [24]. In particular, it requires the production of singlet oxygen (^1^O_2_) that allows the formation of covalent bonds between (hydroxy)proline and histidine residues in adjacent chains [25]. On the other hand, UV irradiation, without riboflavin, may also produce radicals in tyrosine and phenylalanine residues, thus inducing the crosslinking of collagen molecules [26,27]. This may explain the increase of the stiffness when the tissue was exposed to UV, as shown in Figure 2G and confirmed in all patients (data not shown).

Extracellular matrix (ECM) is an essential structural and functional component of biological tissues. ECM accounts, indeed, for their morphological organization, confers them mechanical properties, and is a microenvironmental sensor for adherent cells. Accordingly, any alteration in the composition, the organization or the structure of ECM provokes deep changes in tissue mechanical properties and cell behavior. The control of the ECM stiffness is, thus, pivotal for tissue homeostasis. Moreover, ECM stiffness is commonly altered in numerous diseases, including solid cancers or cardiovascular diseases, and its control is of therapeutic interest [28]. Deciphering the ultra-structural organization of ECM and their mechanical properties is an important step to better understand the pathogenic processes of diseases associated with ECM remodeling and to propose new therapeutic options. In this context, AFM has been extensively used for probing collagen-based tissues [15,16,17,18,19,29], including tendon [30], cartilage [12] and bone [31]. AFM has shown its relevance to provide reliable information regarding their structural and mechanical properties, with the advantages of minimal sample preparation and ability to operate in physiological conditions. In the present study, AFM provided a relevant way to characterize the collagen structure and stiffness in the joint capsule at the nanoscale. On the studied longitudinal cross-sections, our results show an alignment of fibrils in damaged joint capsules. The collagen fibrils were adjacent to each other, had a similar diameter size, and displayed the typical ∼67 nm D-period value for type I collagen. Interestingly, transmission electronic microscopy analysis of transversal cross-sections of the capsule did not reveal any significant difference in the mean diameter of collagen fibrils between normal and diseased capsules [5], suggesting that shoulder instability does not affect the organization of collagen fibrils. Nevertheless, using a small angle light scattering technique, Debski et al. [6]. showed that collagen fibers in the inferior glenohumeral ligament (IGHL) were not highly aligned, although small regions of localized alignment were found. Thus, the collagen organization at the micrometric scale suggests that IGHL is loaded in multiple directions. This random organization implies that during a capsular shift procedure, the ability of the IGHL to transfer loads across its entire insertion into the glenoid/labrum may be significantly altered [6].

The random organization of collagen fibers supports the idea that global treatment of the capsule structure should be efficient to increase the efficiency of surgery. Thermal treatment of unidirectional instability is not performed anymore due to high recurrence rates and complications, including temporary nerve injuries that usually involve the sensory branch of the axillary nerve and rare thermal necrosis of the capsule [3]. A crosslinking procedure may achieve this goal. It has been routinely used in ophthalmology for decades [11]. In joint tissues, crosslinking protocols have been performed on tendons [32], but has not been proposed for capsules yet, to the best of our knowledge. Enzymatic crosslinking protocols affect the whole tendon function, increasing failure load of individual collagen fibrils that paradoxically yield diminished tissue-failure properties [32]. However, contradictory reports have inconsistently correlated crosslink density to tissue stiffness [32]. Our results on the shoulder capsule show that the crosslinking procedure increased mechanical stiffness without modifying the structure and the morphology of collagen fibrils within the damaged capsules and without inducing immediate cell death. Such a capsular modification could be indicated in complement of shoulder stabilization for anterior or posterior instability and could be also useful for patients with very painful multidirectional instability, with or without connective tissue disorders like Ehlers Danlos patients [33]. In addition, crosslinking treatment of the global capsule, increasing its stiffness, may be efficient for patients with abnormally high laxity like hyperlaxity spectrum disorder diseases, such as Ehlers Danlos or Marfan syndrome.

Some limitations emerge from our study. Although we show the effect of UV and riboflavin treatment on tissue stiffness, it is still unknown whether the elastic modulus obtained after crosslinking is comparable with healthy tissue. Moreover, further investigations are needed to identify the clinically relevant conditions for crosslinking treatment. Before starting clinical studies, the long-term efficacy and safety of crosslinking, the combination modality of crosslinking and surgery, the outcomes after crosslinking two or more times, and the efficacy and complications of crosslinking procedures with UV need to be determined [11]. In addition, the effect of UV irradiation and riboflavin was analyzed on small tissue samples due to the small size of the capsule harvested during the surgical procedure. The efficiency of the treatment in the depth of the capsule has not been studied; the question, clearly, deserves further investigation.

## 4. Materials and Methods

### 4.1. Sample Preparation

Articular capsules were collected from five patients undergoing shoulder surgery in the Maussins-Nollet Clinic (Paris, France). Informed consent was obtained from each patient on the day before surgery. Experiments using human samples have been performed in accordance with the Declaration of Helsinki and have been approved by a French Institutional Review Board (Comité de Protection des Personnes Ile de France V). All experiments were performed in accordance with the relevant guidelines and regulations. The Latarjet procedure was performed to stabilize the joint, allowing for the removal of 1 cm^2^ of capsule at the anterior and superior aspect of the detached capsule, that is no more useful in this surgical procedure. Each sample was cut into eight equal pieces of about 1 mm^3^ (Figure 1A). Following the dissection, tissues were distributed fairly among two 96-well tissue culture plates corresponding to UV conditions and control conditions. Samples were incubated in 150 μL of PBS buffer solution with or without riboflavin at various concentrations for 5 min in the dark. The different procedures used for sample preparation are summarized in Table 1. After changing the treatment solution of each well for a new one, articular capsules were incubated for 30 min at room temperature. During this incubation period, the UV condition plate was illuminated by UV beam (λ = 365 nm), while for the control condition the plate was protected from the light (Figure 1B). All treatment solutions were changed every 5 min during this 30 min. After treatment, tissues were washed in PBS for 30 min, embedded in O.C.T. compound (Leica Biosystems, Nanterre, France) and stored at −80 °C until cryosectioning. For each treatment condition, tissues were sectioned with a thickness of 7 µm for biological analysis and 14 µm for structural and mechanical characterizations. The different samples are labelled R_H_, R_M_ and R_L_ according to the high (2.5%), medium (1.0%) or low concentration of riboflavin (0.1%), respectively, with or without UV (see Table 1).

### 4.2. Cell Death Detection

Apoptosis was determined using the in situ cell death detection kit, Fluorescein (Roche-11684795910, Basel, Switzerland). The TUNEL assay was carried out according to the manufacturer’s instructions. Briefly, the articular capsule slices were fixed in paraformaldehyde (3.7% in PBS, pH 7.4) for 20 min and incubated in permeabilization solution (0.1% Triton X-100, 0.1% sodium citrate) for 2 min on ice. Free DNA 3′-OH ends were next labelled with fluorescein-labeled nucleotides (fluorescein-dUTP) in the presence of a Terminal deoxynucleotidyl Transferase (TdT) for 60 min at 37 °C in a humidified atmosphere in the dark.

Tissues incubated in dUTP without TdT enzyme were used as negative control, while positive control had DNA strand breaks induced with recombinant DNase 1 (0.1 mg/mL for 10 min), prior to the labelling procedure. Samples were directly analyzed under fluorescence microscope (excitation wavelength of 450–500 nm and detection in the range of 515–565 nm). DAPI staining was used to check the location of the nucleus (Figure 1C_1_).

### 4.3. Atomic Force Microscopy

Experiments were performed using a commercial AFM (NanoScope VIII MultiMode AFM, Bruker Nano Inc., Nano Surfaces Division, Santa Barbara, CA, USA). The human tissue samples were immobilized on a chemically modified glass slide (Figure 1C_2_) to avoid movement (vertical or lateral) that may generate artifacts during imaging and force curve acquisition. Glass slides (disks, 12 mm of diameter, Thermo Scientific) were cleaned prior to use in a piranha solution (H_2_SO_4_ (98%)/H_2_O_2_ (27%), 3/1, *v*/*v*) and thoroughly rinsed with ultrapure water (Milli-Q, Millipore, France). The glass slides were then incubated in dopamine solution (1 mg/mL dopamine in 10 mM Tris, pH 8.5) at room temperature for 24 h while ensuring a gentle stirring. The glass slides were then rinsed in three different baths (2 min each) of ultrapure water and dried under nitrogen gas flow.

Glass slides, on which the human tissues were cryo-sectioned, were fixed on a steel sample puck using a small piece of adhesive tape. Images were recorded both in the hydrated (PBS buffer) and the dried states at room temperature (20–22 °C). Oxide-sharpened microfabricated Si_3_N_4_ cantilevers were used (SNL-10, Bruker Nano Inc., Nano Surfaces Division, Santa Barbara, CA, USA). The curvature radius of the tips was about 10 nm (manufacturer specifications). All topographic images shown in this paper were flattened using a third-order polynomial to correct surface tilt and to eliminate bow effects.

Force-distance curves were recorded in PBS buffer at room temperature using microfabricated Si_3_N_4_ cantilevers (MLCT from Bruker Nano Inc., Nano Surfaces Division, Santa Barbara, CA, USA). The spring constants of the cantilevers were measured using the thermal noise method, yielding values ranging from 0.025 to 0.0482 N/m. The deflection sensitivity was calibrated by recording the response of the cantilever on a silicon wafer substrate, considered as an infinitely stiff surface. The curvature radius of silicon nitride tips was about 20 nm (manufacturer’s specifications). The same tip was used for all the measurements of the same patient (for all four conditions, without modification of the parameters), which removes the variability that could otherwise have occurred between conditions. A visual control of the tissue was performed using the optical microscope coupled with the AFM instrument to verify tissue integrity and define an area of interest. All force curves were recorded by applying a 1 nN force trigger with a 1 μm ramp size and approach/retraction velocities of 8 µm/s. For each sample, force-distance curves were acquired on at least four areas of the sample. For each area (2 µm × 2 µm), curves were recorded with a force volume parameter set to 64 by 64 pixels for 1 area and 16 by 16 pixels for 3 areas, resulting in a minimum of 1700 curves per sample. For each condition (CTRL, UV, R and R + UV, see Table 1), three different samples were used, so at least 5000 curves were recorded per condition per patient. Since the edge of treated tissues appeared yellow due to the accumulation of riboflavin, only the non-colored internal part was analyzed.

### 4.4. Data Analysis

At least 3 images of 2 µm × 2 µm were acquired per condition and per patient and used to determine the intrinsic characteristics of the collagen fibrils (mean diameter and D-period). The D-period corresponds to a succession of gaps and overlaps along the fibrils, generally around 67 nm for type I collagen. Measurements were obtained from at least 100-line scans obtained on different AFM height images. Box plots were generated by pooling all the data as a function of the condition and patient. Wilcoxon matched paired tests were performed with GraphPad Prism 5 software to compare the characteristics of the collagen fibrils between different conditions for each patient. A *p*-value of < 0.0001 was considered to be significant.

An in-house pyAF (python Atomic Force) software, version 1.5.1, was used to analyse the force-distance curves. To detect the point of contact on the force curves, a linear fit was made using the baseline of the approach curve. A noise threshold was used to shift the fit along the force axis. The noise parameter can be manually adjusted by the user to optimize the detection of the point of contact. The position of the contact point is determined by the intersection between the force curve and this shifted fit. Subsequently, the elasticity was deduced from the Young’s modulus, which was determined from the indentation portion of the curve using the elastic contact model for pyramidal indenters (named Bilodeau model [34]) using the following equation:

(1) $$F=\frac{3}{4}\frac{E\tan\alpha }{\left(1-v^{2}\right)}\delta^{2}$$

where *F* is the measured force, *E* the elastic modulus, *α* the face angle of the pyramid, *δ* the indentation depth, and *ν* the sample’s Poisson’s ratio that was set to 0.5, which corresponds to an incompressible material.

The results are then presented in the form of 2 µm × 2 µm elasticity maps recorded on the 64 by 64 curve experiments where Young’s modulus is displayed as coloured pixels that reflect the tissue stiffness magnitude. Box plots were also generated by pooling all the data from the elasticity maps as a function of the condition and patient. An unpaired t-test with Welch’s correction (which assume no equal SDs) was performed to compare Young’s modulus between conditions (*** *p*-value < 0.0001. A *p*-value of < 0.0001 was considered to be significant). The bottom and top of the boxes represent the first and third quartiles, respectively. The line within the bow corresponds to the second quartile, i.e., the median. The whiskers represent the 5th and the 95th percentiles.

## 5. Conclusions

Glenohumeral joint instability is a frequent disorder of young sportive patients. Its treatment by classical surgical procedures is not efficient and is associated with recurrence. In the present study, we used a crosslinking treatment based on a combination of UV irradiation and riboflavin to reinforce the tissue, which was mainly constituted of type I collagen fibrils. For this purpose, human articular capsules were collected from different patients undergoing shoulder surgery. Results show that the crosslinking procedure changed the biomechanics of diseased capsules by increasing the tissue stiffness while keeping their structural organisation unchanged, and without inducing any significant cell death. Accordingly, the UV/riboflavin crosslinking procedure is a promising way to preserve the functions of collagen-based tissues and to tune their elasticity for clinically relevant experimentation.

## Figures and Tables

**Table 1:** Treatment solution of each plate well according to the associated condition.

Plate	Sample	Treatment	Solution
Control condition	CTRL	Control Riboflavin	PBS 1X
	R_H_	Riboflavin 2.5%	Riboflavin 2.5% in PBS 1X
	R_M_	Riboflavin 1.0%	Riboflavin 1.0% in PBS 1X
	R_L_	Riboflavin 0.1%	Riboflavin 0.1% in PBS 1X
UV condition	UV	Control UV	PBS 1X
	UV + R_H_	Riboflavin 2.5%	Riboflavin 2.5% in PBS 1X
	UV + R_M_	Riboflavin 1.0%	Riboflavin 1.0% in PBS 1X
	UV + R_L_	Riboflavin 0.1%	Riboflavin 0.1% in PBS 1X

## Data Availability

Not applicable.
